# Gender-Specific Trends and Determinants of Daily Smoking in Latvia (2009–2019): A Population-Based Cross-Sectional Study

**DOI:** 10.3390/medicina62061201

**Published:** 2026-06-22

**Authors:** Peteris Apinis, Iveta Bajare, Vilnis Dzerve, Sanda Jegere, Lilian Tzivian, Baiba Kokina, Artis Luguzis, Anda Caksa, Andrejs Erglis

**Affiliations:** 1 Institute of Cardiology and Regenerative Medicine, University of Latvia, LV-1004 Riga, Latvia; peteris.apinis1@gmail.com (P.A.); iveta.bajare@stradini.lv (I.B.); vilnisdzerve@inbox.lv (V.D.); anda.caksa@gmail.com (A.C.); 2 Latvian Centre of Cardiology, Pauls Stradins Clinical University Hospital, LV-1002 Riga, Latvia; sjegere@gmail.com (S.J.); baiba.kokina@gmail.com (B.K.); 3 Faculty of Medicine and Life Sciences, University of Latvia, LV-1004 Riga, Latvia; 4 Institute of Clinical and Preventive Medicine, University of Latvia, LV-1079 Riga, Latvia; liliana.tz@gmail.com; 5 Department of Residency, Riga Stradins University, LV-1007 Riga, Latvia; 6 Faculty of Physics, Mathematics and Optometry, University of Latvia, LV-1004 Riga, Latvia; artis.luguzis@lu.lv

**Keywords:** smoking, physical activity, education level, cardiovascular risk, population-based study, cross-sectional study, Latvia

## Abstract

*Background and Objectives*: Cigarette smoking remains a leading modifiable cardiovascular risk factor. This study aimed to analyze decade trends in daily smoking prevalence and its association with education level and physical activity by gender and age groups in Latvia. *Materials and Methods*: We analyzed data from two distinct waves of a population-based cross-sectional study conducted in Latvia. The study included a stratified random sample of adults aged 25–74 (*N* = 3807 in 2009; *N* = 4070 in 2019). Smoking status and education level were self-reported in both years; physical activity data were collected only in 2019. Multivariable logistic regression identified independent factors associated with smoking. *Results*: From 2009 to 2019, smoking prevalence decreased among men (from 43.6% to 36.0%, *p* < 0.01) and increased among women (from 15.0% to 18.1%, *p* < 0.01). Men and women with higher education were significantly less likely to be daily smokers (2019, Men: odds ratio (OR): 0.48, 95% confidence interval (CI) 0.38–0.59; Women: OR: 0.39, 95% CI 0.29–0.50). Among men, the proportion of daily smokers was higher in physically inactive (39.8%) and active (39.0%) groups compared to the moderately active group (31.4%, *p* < 0.01). However, physical activity level was not a significant predictor of smoking in the multivariable analysis. *Conclusions*: Over the decade, daily smoking prevalence decreased in men but increased in women in Latvia, with education level being a strong independent predictor. Although the proportion of smokers among men differed across physical activity groups, this factor was not an independent predictor of smoking. These findings underscore the need for targeted public health interventions in Latvia, specifically focusing on individuals with lower education levels to reduce the long-term cardiovascular burden.

## 1. Introduction

The European Society of Cardiology identifies smoking as a causal and major modifiable risk factor for cardiovascular disease (CVD) [1]. Global estimates indicate that in 2019, 1.14 billion individuals were current smokers, with tobacco use accounting for 7.69 million deaths and remaining the leading risk factor for mortality among males [2]. Smoking, along with systolic blood pressure, non-high-density lipoprotein cholesterol, diabetes, and body-mass index, accounts for more than half of CVD incidence and 20% of global mortality [3]. At age 50, the absence of these modifiable factors is associated with an estimated life expectancy extension exceeding 10 years [4]. Similarly, smoking cessation at age 40 is associated with a 90% reduction in the excess risk of death [5]. However, only 5.8% of European adults maintain a healthy lifestyle, as assessed by five core health behaviors: physical activity, fruit and vegetable consumption, sleep quality, alcohol consumption, and smoking status [6].

Beyond CVD, smoking is a modifiable risk factor for numerous non-communicable conditions, including lung diseases [7], periodontal disease [8], osteoporosis or osteopenia [9], and erectile dysfunction [10]. Additionally, emerging evidence highlights sex-specific systemic effects of smoking, which predispose women to a higher prevalence of vasomotor menopausal symptoms [11].

Addressing this complex health burden in a population with high cardiovascular risk, such as Latvia, requires a comprehensive understanding of how smoking behaviors intersect with specific socio-demographic and lifestyle determinants. Prior studies have demonstrated that smoking status is associated with education level [12], where higher education correlates with more successful smoking cessation outcomes [13], and physical activity levels [14,15]. In addition to these socio-demographic determinants, smoking is closely associated with broader physiological and lifestyle-related factors. Recent evidence indicates that smokers may exhibit less favorable body composition profiles, including higher fat mass and visceral adiposity, particularly in younger populations [16]. Furthermore, smoking tends to cluster with other unhealthy behaviors, such as increased alcohol and caffeine consumption [16]. These findings suggest that smoking should be considered within a wider behavioral and metabolic context, rather than as an isolated risk factor.

Regular physical activity has a positive impact on cardiovascular health, reducing the relative risk of cardiovascular mortality [17]. It also contributes positively to neurocognitive and mental health, postponing the onset of dementia [18], improving sleep quality [19], and supporting metabolic health and weight management [1]. It also benefits the musculoskeletal system, increasing bone density and reducing fracture risk [20]. Physical activity levels are associated with education level [21].

The primary objective of this study was to analyze 10-year trends in daily smoking prevalence in Latvia (2009–2019), stratified by gender and age. Secondary objectives were to determine the associations between smoking and socio-demographic factors (education level and physical activity) and to assess whether these tendencies align with broader European trends.

## 2. Materials and Methods

### 2.1. Study Design and Patient Population

This study is reported in accordance with the Strengthening the Reporting of Observational Studies in Epidemiology (STROBE) guidelines for cross-sectional studies [22]; the completed STROBE checklist is provided in Supplementary Table S1. We analyzed data from two distinct waves of an ongoing, continuous nationwide population-based cross-sectional study in Latvia, conducted in 2009 and 2019, involving adults aged 25 to 74. In both instances, a target sample of 6000 individuals was selected using a stratified systematic random sampling design. In 2009, the sampling was based on the Latvian Population Register, while in 2019, the Housing Register of the Central Statistical Bureau of Latvia was utilized. The strata were defined by gender (men, women) and age (ten groups with 5-year intervals), with 300 individuals per stratum. To maintain the methodological rigor of the stratified random design, non-respondents were not replaced. The comprehensive participant recruitment, exclusion pathways, and final participation rates for both study waves are detailed in the Section 3.1. The study design and methodology were approved by the Ethics Committee of Research, Institute of Cardiology, University of Latvia (No. 5-28052008, 28 May 2008) and the Ethics Committee of the Institute of Cardiology and Regenerative Medicine, University of Latvia (No. 2-260918, 26 September 2018). More detailed information about the study design has been published previously [23,24].

### 2.2. Data Collection

Data on socio-economic status, education, and lifestyle profile were obtained through face-to-face interviews, following the CINDI Health Monitor Survey principles [25]. Detailed information regarding data collection can be found in previous publications [23,24].

### 2.3. Smoking

Current daily smoking was defined as smoking cigarettes every day for at least one year. Additional smoking-related habits, such as time to the first cigarette after waking up, were assessed exclusively among daily smokers.

### 2.4. Physical Activity

Physical activity levels were categorized based on respondents’ self-reported frequency of activity: (1) physically inactive (sedentary lifestyle, not doing any physical activities); (2) moderately active (walking, exercising, cycling, or swimming 2 to 3 times a week); (3) physically active (running, cycling, attending the gym, or similar activities 3 to 4 times a week); and (4) professional athlete. For the multivariable analysis, physical activity was categorized into three levels: inactive, moderately active, and active. To ensure statistical stability and minimize potential misclassification bias, respondents who identified as professional athletes (*N* = 9) were merged into the ‘active’ category for all predictive modeling.

### 2.5. Alcohol Consumption

Alcohol consumption was assessed based on participants’ self-reported frequency of alcohol intake during the previous year. The response categories were: (1) never; (2) once a month or less; (3) 2–4 times a month; (4) 2–3 times a week; and (5) 4 or more times a week.

### 2.6. Education

Education levels were classified into three categories: (1) primary education (primary school education or lower); (2) secondary education (general secondary, vocational secondary, or first-level professional education); and (3) higher education (bachelor’s degree or higher).

### 2.7. Statistical Analysis

Data analysis was performed using Statistical Package for Social Sciences (IBM SPSS, Armonk, NY, USA) version 29.0.0. Descriptive statistics were calculated for all study variables, stratified by survey year (2009 vs. 2019) and gender. Group distribution for categorical variables (smoking status, physical activity, education) was presented as counts and percentages, while continuous variables (e.g., age) were summarized as medians with interquartile ranges (IQR), as none of the continuous variables showed a normal distribution.

Differences between groups (2009 vs. 2019; men vs. women) were assessed using the Mann–Whitney U test for continuous variables and the Chi-square test or Fisher’s exact test for categorical variables.

To identify factors associated with daily smoking, a two-step analysis was performed. First, a univariate analysis was conducted to assess the relationship between demographic variables and daily smoking. To check for potential confounders, a Directed Acyclic Graph (DAG) was built, yielding a minimal adjustment set of age and gender (Supplementary Figure S1). Based on the univariate relationships and our epidemiological knowledge, additional factors were included in the multivariable logistic regression model. Multivariable logistic regression was fitted to identify independent predictors of daily smoking for both study waves. Additionally, a separate sensitivity analysis was performed for the 2019 dataset to evaluate the impact of physical activity. In this specific model, physical activity was treated as a 3-level categorical variable (inactive, moderately active, active). Results were reported as odds ratios (ORs) with corresponding 95% confidence intervals (95% CIs). An alpha level of 0.05 was used for all statistical tests.

## 3. Results

### 3.1. Characteristics of the Study Population

**Figure 1:**
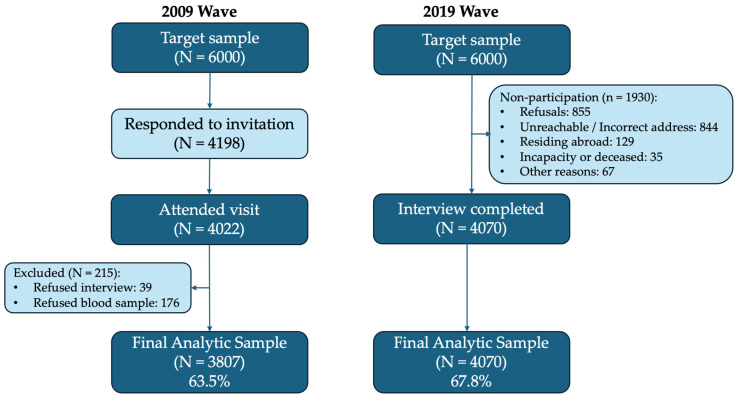
Study flow chart illustrating participant recruitment, non-participation reasons, and final analytical samples for the 2009 and 2019 survey waves.

The study analyzed data from a total of 7877 respondents: 3807 in 2009 (63.5% of the target sample) and 4070 in 2019 (67.8% of the target sample). The comprehensive participant recruitment and exclusion pathways for both waves are systematically illustrated in Figure 1. In the 2009 study, 4198 individuals initially responded to the invitation, and 4022 individuals subsequently attended the clinical visit. From these, 215 subjects were excluded due to an incomplete protocol (176 missing blood samples and 39 refused to complete the full interview), resulting in a final analytic sample of 3807 (63.5%) individuals. Conversely, the 2019 study wave utilized face-to-face home interviews, where a total of 4070 individuals (67.8%) successfully completed the questionnaire to form the final analytic sample. The remaining 1930 non-respondents from this wave were accounted for by explicit refusals (*n* = 855; 14.3%), unreachable individuals or incorrect addresses (*n* = 844; 14.1%), subjects residing abroad (*n* = 129; 2.2%), incapacity due to illness or deceased status (*n* = 35; 0.6%), and other technical reasons (*n* = 67; 1.1%).

The median age of the total population was 52.0 years (IQR = 40.0–63.0); 43.1% were male and 56.9% were female. Detailed demographic and lifestyle characteristics of the study samples, stratified by gender and year, are presented in Table 1. The study population in 2009 was significantly older than in 2019 (median age 54.0 years [IQR = 43.0–64.0] vs. 50.0 years [IQR = 37.0–62.0], *p* < 0.01). This difference was largely driven by a higher number of participants and an improved response rate in the 25–34 age group during the 2019 study. Additionally, the proportion of female respondents significantly decreased from 63.9% in 2009 to 50.5% in 2019 (*p* < 0.01), owing to a substantial increase in male participation in the second study wave.

Over the decade, the prevalence of daily smoking exhibited opposing trends by gender. This was characterized by a significant increase among women (from 15.0% to 18.1%, *p* < 0.01) and a significant decrease among men (from 43.6% to 36.0%, *p* < 0.01). The male-to-female smoking prevalence ratio decreased from 2.91 in 2009 to 1.99 in 2019.

Statistically significant differences were also observed regarding education level, family status, and alcohol consumption. In terms of education, the proportion of men with secondary education increased, while the proportion with higher education decreased. Conversely, among women, the proportions of those with primary and higher education increased, whereas the share of those with secondary education declined. Alcohol consumption patterns revealed a decrease in the proportion of lifetime abstainers (“never” users), declining from 50.8% to 20.7% in men and from 67.7% to 28.5% in women.

Physical activity was evaluated exclusively in the 2019 study. Male respondents were predominantly physically active, while females were largely moderately active. Specifically, 48.6% of men and 29.4% of women reported being physically active, whereas 41.2% of men and 57.4% of women reported a moderately active lifestyle. Only 10.0% of men and 13.0% of women reported being physically inactive.

### 3.2. Smoking Patterns Among Study Participants

Comparison of smoking-related parameters in 2009 and 2019 is summarized in Table 2, while detailed comparisons across age groups for men and women are presented in Table 3. In both study years, the lowest smoking prevalence was consistently observed in the oldest age group (65–74 years) for both genders. Conversely, the highest prevalence was found in the 25–34 age group for both men and women in 2009, shifting to the 35–44 age group in 2019.

In the male population, the overall decrease in smoking prevalence was driven by reductions across almost all age groups, with the most prominent decline observed in the 25–34 age group (from 54.0% to 32.2%) and the smallest difference in the 65–74 age group. Conversely, the increasing prevalence among females was attributed to higher rates in three specific age groups: 35–44, 55–64, and 65–74 years. In contrast, a favorable change was observed in younger women (aged 25–34), where smoking prevalence declined from 28.0% to 23.3%. Significant changes in the age distribution of smokers were observed between the two study years for both men (*p* = 0.03) and women (*p* < 0.01).

Regarding smoking habits, the median age of smoking initiation decreased significantly in both men (from medians of 18.0 to 17.0 years, *p* < 0.01) and women (from medians of 20.0 to 18.0 years, *p* < 0.01). Furthermore, there was a significant increase in the proportion of smokers who consume their first cigarette within 30 min of waking up (men: 46.3% vs. 54.9%; women: 37.6% vs. 45.6%), with no statistically significant differences observed between age groups within the same study year.

Finally, fewer respondents expressed willingness to quit smoking in 2019 compared to 2009, a trend that was consistently observed across all age groups.

### 3.3. Relationship Between Smoking and Education Level

The association between smoking and education level is shown in Table 4. Overall, higher education was associated with a lower proportion of smokers. Comparing 2009 and 2019, significant shifts in smoking patterns relative to education were observed in both genders (*p* < 0.01). In men, a significant decrease in the proportion of daily smokers was observed in the secondary education group (from 49.2% to 38.4%) and the higher education group (from 34.5% to 17.7%). In contrast, among men with primary education, the percentage actually increased (from 42.0% to 55.0%). Among women, a decrease in the proportion of smokers was observed only in the higher education group (from 13.2% to 8.9%). In contrast, smoking rates increased among women with secondary education (from 16.5% to 21.7%) and nearly tripled among those with primary education (from 12.2% to 33.2%).

### 3.4. Relationship Between Smoking and Alcohol Consumption

A dose–response relationship was observed between alcohol consumption and smoking (Table 4). Higher alcohol consumption was significantly related to a higher proportion of smokers (*p* < 0.01), with the exception of men in 2009, where the difference was not statistically significant (*p* = 0.80). For instance, in 2019, the smoking percentage among men who never consumed alcohol was 24.9%, compared to 58.2% among those consuming alcohol ≥ 4 times a week. A similar pattern was observed in women, where the smoking percentage was lowest among never-drinkers (14.0% in 2009 and 14.9% in 2019) and significantly higher among frequent consumers.

### 3.5. Relationships Between Smoking and Physical Activity

The relationship between smoking and physical activity levels in the 2019 study differed by gender (Table 4). A statistically significant relationship was found in men (*p* < 0.01), but not in women (*p* = 0.10). Among male respondents, the proportion of smokers was highest in the physically inactive group (39.8%) and the physically active group (39.0%), whereas those reporting a moderately active lifestyle had a lower smoking percentage (31.4%). Professional athletes represented the smallest absolute number of smokers, though the group size was too small for meaningful comparison. Although the lowest smoking percentage was recorded among professional athletes, the sample size for this group was very small. In women, despite a slightly higher smoking rate among physically active respondents (21.2%), no statistically significant relationship was observed (*p* = 0.10).

### 3.6. Distribution of Physical Activity Levels Among Daily Smokers Stratified by Age and Education

**Figure 2:**
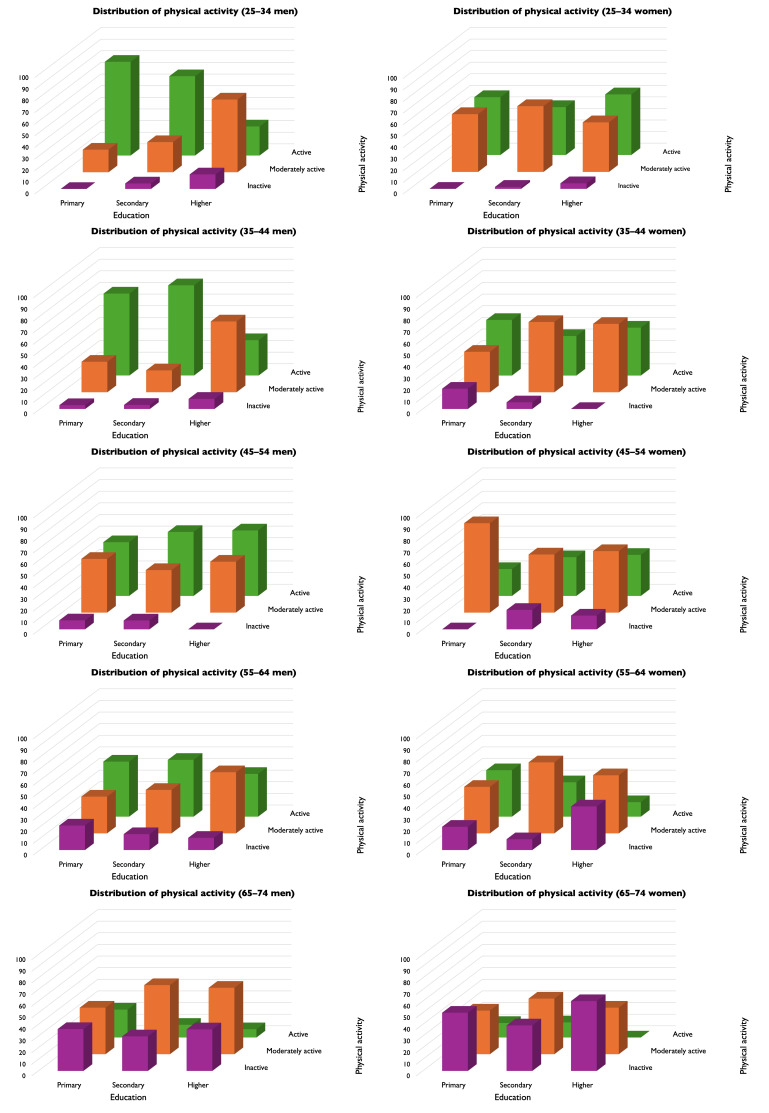
Distribution of physical activity levels among daily smokers stratified by age, gender, and education level.

Figure 2 and Table 5 present the distribution of physical activity levels among daily smokers, stratified by age and education. In men, physical activity levels differed significantly by education in the 35–44 (*p* < 0.01) and 65–74 age groups (*p* < 0.01). Men aged 25–44 with primary or secondary education were predominantly physically active (68.2–80.6%), whereas those with higher education were predominantly moderately active. In the 65–74 age group, moderate activity was the most frequent status, with the proportion of physically inactive men reaching 36.0% among those with primary education and 29.7% among those with secondary education.

In women, no significant relationships between education and physical activity were observed for most age groups (*p* > 0.05). Female smokers were predominantly moderately active across most categories. The only exception was women aged 25–34 with higher education, who were predominantly physically active (52.4%), although the difference based on education was not statistically significant (*p* = 0.26). A significant difference was observed in the 65–74 age group (*p* = 0.02), where physical inactivity was the dominant status for women with primary and higher education.

### 3.7. Multivariable Analysis of Factors Associated with Daily Smoking

The results of the multivariable logistic regression analysis, identifying independent factors associated with daily smoking for men and women, are presented in Table 6. In men, age was a consistent protective factor in both study years; for every one-year increase in age, the odds of being a daily smoker declined slightly (2009: OR 0.97, 95% CI 0.96–0.99; 2019: OR 0.98, 95% CI 0.97–1.00). Education emerged as a significant determinant in the 2019 study, where a higher education level was associated with significantly lower odds of smoking (OR 0.48, 95% CI 0.38–0.59). However, in 2009, education was not significantly associated with being a daily smoker. Alcohol consumption demonstrated a significant association with smoking in men in both years, though the direction of the association differed. In 2019, alcohol use showed an increased risk (OR 1.30, 95% CI 1.17–1.46), whereas in 2009, an inverse association was observed (OR 0.71, 95% CI 0.58–0.87).

Among women, education level was the strongest independent predictor of smoking status in both 2009 and 2019. Higher education was significantly associated with lower odds of smoking (2009: OR 0.48, 95% CI 0.32–0.72; 2019: OR 0.39, 95% CI 0.29–0.50). While age was not a significant factor for women in 2009, it became significant in 2019, following the same pattern as in men (OR 0.98, 95% CI 0.97–0.99). In contrast to men, alcohol consumption did not show a statistically significant association with smoking in the multivariable model for women in either study year.

To validate the stability of our findings, we performed a sensitivity analysis for the 2019 data by including physical activity as an additional covariate (Table 7, Graphical abstract). In this model, physical activity was analyzed as a three-level categorical variable (inactive, moderately active, and active), with professional athletes being merged into the ‘active’ group due to their small sample size. Physical activity was not a statistically significant predictor of daily smoking for either gender. Importantly, the inclusion of this variable did not alter the significance or magnitude of the associations with other independent factors, confirming the robustness of the primary model.

## 4. Discussion

This study analyzed the 10-year trends of daily smoking prevalence in Latvia between 2009 and 2019. The data demonstrate a divergence between genders, characterized by a significant decrease in smoking prevalence among men and an increase among women, specifically in older age cohorts. Education level emerged as a strong independent predictor of daily smoking for both genders, with lower odds of smoking observed among individuals with higher education and an increasing trend among those with primary education. Regarding lifestyle factors, physical activity level was not a statistically significant predictor of daily smoking in the multivariable analysis. Conversely, alcohol consumption displayed inconsistent trends over the decade, showing an inverse association with smoking among men in 2009 but emerging as a significant independent risk factor in 2019. These distinct gender- and socioeconomic-specific shifts suggest a potential benefit for integrating population-wide public health interventions with complementary, targeted socioeconomic strategies.

### 4.1. Trends in Smoking Prevalence

The significant decline in daily smoking observed among Latvian men aligns with the broader epidemiological patterns reported across European regions, where male smoking prevalence has demonstrated a steady decrease over recent decades [2,26]. This regional reduction is consistent with the Global Burden of Disease analysis, which documents significant declines in age-standardized male smoking prevalence across Western, Central, and Eastern Europe between 1990 and 2019, alongside a 27.5% global decrease [2]. According to Eurostat data, the long-term implementation of tobacco control policies resulted in an average daily smoking prevalence of 22.3% among men in the European Union (EU); however, the male daily smoking prevalence observed in Latvia, despite the documented decline to 36.0% in 2019, remains substantially higher than this EU average and exceeds the rates reported in neighboring Baltic states such as Lithuania (29.1%) and Estonia (25.2%) [26]. The daily smoking prevalence observed among Latvian women demonstrated a slight increase to 18.1% in 2019, positioning it above the average European Union (EU) female prevalence of 14.8% [26]. While female tobacco use has been stabilizing or declining globally, this upward trajectory in Latvia aligns with the concurrent increases documented by Eurostat between 2014 and 2019 in several other EU member states, including Hungary, Germany, and Slovakia [2,26]. Notably, this female cohort elevation is substantially less pronounced than the concurrent decline documented among Latvian men over the same decade, leading to an asymmetrical dynamic where the narrowing of the male-to-female smoking prevalence ratio—from 2.91 in 2009 to 1.99 in 2019—is primarily driven by the rapid reduction in male smoking rather than an explosive escalation among females. In the TackSHS survey across 12 European countries, the average male-to-female ratio was 1.46, with England reporting a ratio as low as 1.04, while Latvia demonstrated the highest disparity in that cohort [27]. This disparity may indicate that Latvia is currently in an earlier stage of the tobacco epidemic compared to Western European nations; however, as these gender gaps slowly narrow across the continent, European demographic projections emphasize that the converging progression of such lifestyle-attributable risk trends fundamentally shapes future sex-specific mortality, wherein the delayed impact of the smoking epidemic among women acts as a temporary brake on their life expectancy gains, thereby narrowing the historical survival advantage over men [28].

### 4.2. Nicotine Dependence and Motivation to Quit

A concerning finding in this study is the increase in markers of nicotine dependence, accompanied by a declining willingness to quit. Although overall smoking prevalence declined over the decade, the proportion of daily smokers presenting with high nicotine dependence expanded significantly, while the self-reported desire to undergo cessation efforts demonstrated notable reductions across both sexes. In comparison, these descriptive patterns align with recent European data from the International Tobacco Control (ITC) Spain Survey, which documented that less than half of current smokers (45.6%) maintained any intention to quit [29]. Similarly, the observed trajectories closely mirror longitudinal evidence from the German Study on Tobacco Use (DEBRA), which likewise reported a progressive decline in cessation motivation among daily smokers within a comparable timeframe [30]. While the cross-sectional design of this study precludes tracking individual behavioral trajectories, these concurrent shifts—rising physical dependence and eroding motivation—suggest a distinct alteration in the aggregate profile of the remaining smoking population in Latvia. At a population level, this trajectory potentially aligns with the “hardening hypothesis,” presenting a contrast to the “softening” trends documented in several Western and Northern European populations where decreasing prevalence typically coincides with lower aggregate dependency levels [31,32,33]. These findings may suggest that, at a population level, the remaining smokers in Latvia are becoming more deeply addicted and less responsive to standard interventions, indicating that conventional cessation advice may no longer be sufficient.

### 4.3. Impact of Public Health Interventions

The observed general decline in male smoking prevalence coincides with Latvia’s active implementation of the World Health Organization (WHO) MPOWER framework over the decade, including expanded smoke-free legislation, regular increases in tobacco excise duties, and the introduction of visual health warnings on packaging [34]. While these regulatory strategies, accompanied by broad public educational campaigns, effectively contributed to national progress among men, they often lacked precise audience targeting. Crucially, no tailored prevention or cessation campaigns were directed at individuals with primary education or lower socioeconomic status. This strategic omission likely contributed to the poor outcomes and the increasing prevalence observed within this demographic, despite the overall national progress.

Furthermore, the documented stagnation in the general population’s motivation to quit highlights a critical public health policy gap. While macro-level legislative restrictions and structural barriers are highly effective at deterring smoking initiation among younger cohorts, they fail to provide adequate, proactive clinical support for cessation among heavily dependent, established smokers. Consequently, these findings emphasize that gender-neutral and socio-economically undifferentiated approaches have reached a point of diminishing returns in Latvia, necessitating a transition toward highly targeted public health interventions.

### 4.4. The Role of Education

Over the studied decade, our findings demonstrate a widening socio-economic gradient in daily smoking prevalence in Latvia, marked by a pronounced educational polarization. While daily smoking rates progressively declined among highly educated cohorts of both genders, a concerning upward trajectory emerged within lower-educated strata, most notably among women with primary education. These findings are consistent with international trends, confirming that while smoking prevalence is declining among the highly educated, it remains high or is increasing among those with lower educational levels [35]. This growing disparity confirms that while anti-smoking campaigns and macro-level public health messages have effectively reached and modified behaviors within more privileged groups, they have been insufficient for socio-economically disadvantaged populations.

This widening divide reflects a broader epidemiological phenomenon described by the inverse equity hypothesis, which posits that health-seeking behaviors and public health interventions permeate highly educated groups first, leaving lower socio-economic strata disproportionately exposed to long-term health risks [36]. Furthermore, the changing role of education over time carries structural implications. While education level did not act as a statistically significant independent predictor of daily smoking among men in the 2009 baseline multivariable model, its emergence as a dominant determinant in 2019 suggests that Latvia’s health transition has rapidly accelerated. Tobacco consumption habits have increasingly polarized strictly along socio-educational lines, highlighting an urgent need for equity-focused, socio-economically tailored interventions.

### 4.5. Smoking and Alcohol Consumption

Regarding the male population, our study revealed contrasting associations between smoking and alcohol consumption across the two study waves, transitioning from an inverse relationship in 2009 to a strong direct association in 2019. This observed reversal is noteworthy and likely reflects genuine changes in lifestyle clustering within Latvian society over the decade. It is possible that in 2009, during a period of significant economic recession, individual consumption patterns were uniquely affected by competing socio-economic pressures. It has been previously observed that economic crises can trigger complex shifts in substance use; while increased psychological stress may drive higher consumption, significant budgetary constraints can simultaneously limit the ability to maintain multiple unhealthy habits at once [37]. By 2019, however, smoking behavior became more closely linked to higher alcohol intake, reflecting a more traditional clustering of unhealthy lifestyle factors often observed in stable market economies. In contrast to men, alcohol consumption did not show a statistically significant association with smoking in the multivariable model for women in either study year. This suggests that the behavioral drivers of smoking may differ by gender, highlighting the need for gender-specific public health approaches. The association observed in men in 2019 aligns with extensive international research demonstrating that tobacco and alcohol use frequently co-occur and reinforce each other through shared neurobiological and behavioral mechanisms [38,39]. Furthermore, recent large-scale population studies indicate that this clustering remains robust even when accounting for psychological distress, with smokers showing significantly higher odds of engaging in high-risk alcohol consumption [40]. This synergistic relationship suggests that public health interventions addressing cardiovascular risk factors should not be isolated; rather, smoking cessation programs might be more effective if they simultaneously address alcohol consumption patterns, as a recent systematic review indicates that alcohol users are significantly less likely to successfully quit compared to abstainers [41].

### 4.6. Physical Activity and Smoking

In our study, no association was found between physical activity and smoking, although the proportion of smokers differed across the various physical activity groups in men. The high proportion of smokers among physically active men in Latvia contrasts with established global patterns. Acar and colleagues, in a recent national population-based study in Germany, observed that higher physical activity levels are strongly associated with lower odds of current smoking, reduced daily cigarette consumption, weaker cravings, and significantly higher motivation to stop smoking [15]. Similarly, large-scale social surveys in China support the hypothesis that regular physical exercise inhibits smoking behavior, primarily operating through the enhancement of mental health and social support networks [42]. The epidemiological discrepancy likely reflects differences in the stage of the tobacco epidemic; according to Eurostat data, daily smoking prevalence in Latvia remains significantly higher than in Western European nations [26]. It is plausible that in transitional regions characterized by a high smoking prevalence, tobacco use is not yet as heavily marginalized as in more advanced tobacco control settings. This dynamic could potentially allow smoking behaviors to coexist with recreational physical activities before a broader healthy lifestyle clustering fully establishes itself. This cultural context helps explain why the high smoking rates observed in our descriptive data are primarily driven by confounding factors, such as education and alcohol use, rather than by physical activity itself. Once these variables are accounted for in the multivariable model, physical activity loses its independent predictive power, indicating that the observed patterns are a result of lifestyle clustering rather than a direct link between exercise and smoking behavior.

### 4.7. Strengths and Limitations

The main strength of this study is its large, representative population-based sample and the use of the same standardized methodology across two time points a decade apart, allowing for a robust comparison of smoking trends in Latvia.

However, several limitations must be acknowledged. First, the two-wave cross-sectional design of the study limits the ability to establish causal relationships or track individual behavioral trajectories over time, thereby precluding direct evidence for individual-level tobacco hardening. For instance, the unexpected inverse association between alcohol consumption and smoking observed in men in 2009 might be a result of reverse causality rather than a protective effect. Furthermore, as seen from our models, family status became statistically significant in men in 2019. Although in factor analysis these variables did not display potential collinearity (Variance Inflation Factor, VIF = 1.06 for family status and VIF = 1.04 for alcohol consumption), some high intercorrelations among these variables, specifically in 2019, can still be assumed.

Second, relying on self-reported data introduces the risk of recall or social desirability bias, where participants may underreport unhealthy behaviors (like smoking) or overreport physical activity to appear healthier or meet social expectations.

Third, demographic and environmental variations between the two study periods must be taken into account. The 2009 sample included a higher proportion of women and older participants, whereas the 2019 survey achieved a better response rate among men. Although the large sample sizes provide robust data, these variations in sample composition could still slightly influence the comparison of trends. Furthermore, the study setting must be contextualized within Latvia’s shifting socioeconomic landscape. The 2009 wave was executed during a period of severe economic recession in the Baltic states, which profoundly influenced public psychological stress and purchasing power, potentially altering baseline substance use behaviors compared to the more stable economic climate of 2019. Consequently, caution should be exercised when extrapolating these precise trajectory magnitudes to Western European frameworks or larger, multi-ethnic populations with different economic stabilities, regulatory environments, and tobacco taxation histories.

Fourth, non-response and population boundaries remain a challenge. Individuals with the unhealthiest lifestyles might be less likely to participate in health surveys. Additionally, the sample is restricted to non-institutionalized adults; transient, marginalized, or institutionalized populations (such as hospital inpatients or prison populations) without stable addresses were inherently excluded. Given that these omitted subgroups typically exhibit lower health literacy and significantly higher rates of tobacco use, our findings may slightly underestimate the absolute national burden of daily smoking.

In addition, despite adjusting for multiple covariates, the possibility of residual confounding remains, as we did not account for other relevant factors such as psychological health or baseline nicotine dependence levels.

Finally, while this study provides valuable insights into the macroeconomic and demographic trajectories over a ten-year interval, it is important to acknowledge that our epidemiological findings demonstrate what trends occurred, rather than explicitly explaining why they happened. Several significant societal and structural shifts during this decade likely contributed to the opposing trends observed between genders. First, the dramatic evolution of the tobacco marketplace between 2009 and 2019, characterized by the increasing availability and popularity of e-cigarettes and alternative nicotine products, may have altered traditional cigarette consumption patterns in ways our data could not capture. Second, the observed increase in smoking among women may be partially driven by changing socio-cultural dynamics—often associated with “women’s liberation”—where women in Latvian society have increasingly assumed traditionally male-dominated roles, occupations, and, concurrently, associated lifestyle habits. Lastly, the differential, group-specific impact of evolving national tobacco control programs and taxation history over this decade might also play a crucial role. Additional mixed-methods and longitudinal research is urgently needed to explore these specific contextual factors and establish their causal relationships with the trends identified. Ultimately, while our study illustrates what population-level shifts occurred, these methodological boundaries necessitate careful consideration regarding the generalizability of our findings.

### 4.8. Implications for Public Health Policy

The findings of this study demonstrate that relying primarily on opportunistic clinical counseling during routine medical visits is insufficient to address the shifting tobacco epidemic in Latvia, highlighting a critical need to transition toward structured, multi-level public health interventions. This strategic shift directly aligns with the recently adopted “Action Plan for the Improvement of Cardiovascular Health 2026–2027” in the Republic of Latvia [43]. At the population level, this national framework prioritizes wide-reaching health promotion measures, including modifying lifestyle habits, educating the public on self-screening, and encouraging regular self-monitoring. Crucially, at the clinical level, the plan complements these efforts by mandating the systematic implementation of the SCORE2 risk estimation algorithm within primary care, where determining smoking status serves as a key screening parameter. Because smoking status is a heavily weighted variable that dramatically escalates SCORE2 risk scores, these planned screening tools and educational campaigns must be proactively tailored to reach lower socioeconomic and primary education demographics, who, as our study demonstrates, carry a disproportionate burden of tobacco-attributable risk. Furthermore, public health strategy must transition from broad, low-intensity anti-smoking slogans to targeted, socio-culturally sensitive interventions. For the high-risk, lower-educated female demographic identified in our study, standard written warnings or digital campaigns may be less effective; instead, preventive frameworks should integrate community-based outreach and accessible primary care support that explicitly addresses the unique psychosocial stressors driving tobacco use in this cohort. Concurrently, for the male population, clinical protocols should actively move away from treating smoking and harmful alcohol consumption as isolated behavioral issues. Given the strong lifestyle risk clustering demonstrated in our 2019 data, primary care interventions must adopt integrated screening and dual-cessation counseling models, as unaddressed alcohol intake remains a primary driver of smoking relapse. Ultimately, to maximize the clinical impact of these coordinated screening and promotional activities, the healthcare system should ideally transition from merely identifying risk to providing comprehensive support, combining intensive behavioral therapy with accessible pharmacological cessation aids. Crucially, translating these macro-level policies into effective clinical outcomes requires a paradigm shift toward integrated, multilevel, and multidisciplinary management pathways. In the context of contemporary cardiology practice, optimizing smoking cessation interventions cannot rely solely on the brief counseling provided by physicians during routine visits. Evidence highlights the critical need for exploring the role of advanced practice nurses in cardiology, as specialized nursing professionals are uniquely positioned to deliver intensive behavioral counseling, systematically track long-term cessation compliance, and bridge the gap between primary care and specialized cardiovascular clinics [44]. Furthermore, because smoking behaviors frequently cluster with other chronic cardiovascular pathologies, tobacco cessation strategies must be structurally embedded within broader multidisciplinary care in heart failure services [45]. An integrated approach—utilizing the collaborative expertise of cardiologists, advanced practice nurses, nutritionists, and rehabilitation specialists—ensures that interconnected lifestyle risk factors are addressed simultaneously rather than in isolation. Ultimately, developing multidisciplinary management of heart failure and high-risk cardiovascular cohorts through formalized care networks allows for a more holistic, patient-centered mitigation of risk, substantially reducing readmission rates and improving the overall prognosis of patients navigating complex behavioral dependencies [46]. Consequently, transitioning from fragmented lifestyle advice to these cohesive, nurse-led, and multidisciplinary clinical frameworks is essential to operationalize Latvia’s national cardiovascular objectives successfully.”

Finally, to evaluate the long-term impact of health policies and accurately track evolving cardiovascular risk profiles, maintaining a robust national monitoring infrastructure is critical. While massive regional databases often aggregate heterogeneous data collected via varying national methodologies, Latvia’s scale permits the execution of tightly controlled, highly standardized, and strictly population-representative cross-sectional surveys. Continuing this established tracking framework with a subsequent wave in 2029 is essential to capture true behavioral shifts, assess the impact of novel tobacco products, and guide data-driven cardiovascular prevention.

## 5. Conclusions

This study demonstrates that Latvia is undergoing a critical socio-epidemiological transition in smoking prevalence, marked by a distinct gender and socioeconomic polarization. While macro-level tobacco control measures have coincided with a significant decline in smoking prevalence among men and highly educated cohorts, their impact has been less effective among socioeconomically disadvantaged populations. Notably, low educational attainment has emerged as a primary driver of the concerning upward smoking trajectory observed specifically among women. Synthesizing these demographic shifts indicates a clear structural transition toward “population hardening”. The remaining daily smoking population of both sexes exhibits objective signs of deeper nicotine dependence combined with a substantial erosion of cessation motivation. Furthermore, the distinct behavioral risk clustering—where alcohol consumption strongly predicts smoking status strictly in men, while leisure-time physical activity consistently demonstrates no independent, statistically significant association with smoking across either gender—confirms that the drivers of persistent tobacco use are inherently sex-specific. Ultimately, these findings prove that traditional, undifferentiated public health approaches have reached a point of diminishing returns. To effectively mitigate the national cardiovascular burden, prevention strategies must pivot toward integrated, equity-focused, and multidisciplinary clinical interventions tailored to specific socio-educational and gender risk profiles.

## Figures and Tables

**Table 1:** Socio-demographic and lifestyle characteristics of the study population, by gender and year.

Variables	Men 2009 (*N* = 1376)	Men 2019 (*N* = 2016)	*p*-Value 2009 vs. 2019	Women 2009 (*N* = 2431)	Women 2019 (*N* = 2054)	*p*-Value 2009 vs. 2019
Age (years), Median (25–75%), min; max	55.0 (43.0–65.0), 25; 74	50.0 (37.0–62.0), 25.0; 74.0	<0.01	53.0 (43.0–64.0), 25; 74	50.0 (37.0–62.0), 25; 74	<0.01
Age groups (years), *N* (%)						
25–34	137 (10.0)	385 (19.1)	<0.01	225 (9.3)	417 (20.3)	<0.01
35–44	236 (17.2)	399 (19.8)		461 (19.0)	398 (19.4)	
45–54	313 (22.7)	389 (19.3)		592 (24.4)	405 (19.7)	
55–64	323 (23.5)	428 (21.2)		591 (24.3)	420 (20.4)	
65–74	367 (26.7)	415 (20.6)		562 (23.1)	414 (20.2)	
Current daily smoker, N (%)	600 (43.6)	725 (36.0)	<0.01	364 (15.0)	371 (18.1)	<0.01
Education level, *N* (%)						
Primary	176 (13.0)	271 (13.4)	<0.01	962 (40.8)	953 (46.4)	<0.01
Secondary	791 (58.5)	1292 (64.1)		1239 (52.6)	903 (44.0)	
Higher	386 (28.5)	453 (22.5)		156 (6.6)	198 (9.6)	
Family status, *N* (%)						
Married/lives with partner	1058 (77.9)	1349 (66.9)	<0.01	1465 (60.8)	1243 (60.5)	<0.01
Divorced	120 (8.8)	221 (11.0)		342 (14.2)	264 (12.9)	
Widowed	39 (2.9)	47 (2.3)		363 (15.1)	226 (11.0)	
Single	142 (10.4)	399 (19.8)		241 (10.0)	321 (15.6)	
Alcohol consumption. *N* (%)						
Never	556 (50.8)	418 (20.7)	<0.01	1164 (67.7)	585 (28.5)	<0.01
≤once a month	393 (35.9)	641 (31.8)		494 (28.7)	909 (44.3)	
2–4 times a month	94 (8.6)	632 (31.3)		42 (2.4)	469 (22.8)	
2–3 times a week	52 (4.7)	270 (13.4)		19 (1.1)	83 (4.0)	
≥4 times a week	0 (0.0)	55 (2.7)		0 (0.0)	8 (0.4)	
Physical activity, *N* (%)						
Inactive	NA	201 (10.0)	NA	NA	267 (13.0)	NA
Moderately active	NA	830 (41.2)		NA	1180 (57.4)	
Active	NA	979 (48.6)		NA	604 (29.4)	
Professional athlete	NA	6 (0.3)		NA	3 (0.1)	

Results are number (%), or median (interquartile range). Max—maximum; min—minimum; *N*—number; NA—not available.

**Table 2:** Comparison of smoking-related characteristics in daily smokers, by gender (2009 vs. 2019).

Variables	Men 2009 (*N* = 1376)	Men 2019 (*N* = 2016)	*p*-Value 2009 vs. 2019	Women 2009 (*N* = 2431)	Women 2019 (*N* = 2054)	*p*-Value 2009 vs. 2019
Smoking starts (years), Median (25–75%), min; max	18.0 (16.0–20.0), 7.0; 57.0	17.0 (16.0–19.0), 7.0; 54.0	<0.01	20.0 (18.0–25.0), 12.0; 56.0	18.0 (16.0–21.0), 8.0; 62.0	<0.01
First cigarette within 30 min of waking, *N* (%)	278 (46.3)	388 (54.9)	<0.01	137 (37.6)	169 (45.6)	0.03
Would like to quit smoking, *N* (%)	255 (60.7)	356 (49.1)	<0.01	177 (65.8)	193 (52.0)	<0.01

Results are number (%), or median (interquartile range). *N*—number.

**Table 3:** Smoking-related characteristics in men and women by age group in 2009 and 2019.

	2009					*p*-Value	2019					*p*-Value
Age Group	25–34	35–44	45–54	55–64	65–74		25–34	35–44	45–54	55–64	65–74	
Men												
Smoking start age, Median (25–75%)	17.0 (15.5–19.0)	18.0 (16.0–20.0)	18.0 (17.0–20.0)	18.0 (16.0–20.0)	19.0 (16.0–21.0)	<0.01	16.0 (15.0–18.0)	17.0 (15.0–18.0)	18.0 (16.0–19.0)	18.0 (16.0–20.0)	18.0 (16.0–20.0)	<0.01
Current daily smoker, *N* (%)	74 (54.0)	119 (50.4)	155 (49.5)	154 (47.7)	98 (26.7)	<0.01	124 (32.2)	169 (42.4)	160 (41.1)	169 (39.5)	103 (24.8)	<0.01
First cigarette within 30 min of waking, *N* (%)	36 (48.6)	57 (47.5)	79 (51.0)	68 (44.2)	38 (38.8)	0.39	62 (50.0)	84 (49.7)	100 (62.5)	93 (55.0)	59 (57.3)	0.14
Would like to quit smoking, *N* (%)	46 (70.8)	59 (60.8)	77 (62.1)	58 (58.0)	24 (44.4)	0.06	83 (56.5)	94 (51.6)	96 (53.0)	86 (47.3)	48 (42.9)	0.20
Women												
Smoking start age, Median (25–75%)	18.0 (16.0–20.0)	19.5 (18.0–25.0)	20.0 (18.0–26.0)	20.0 (18.0–25.0)	27.0 (19.8–35.0)	<0.01	17.0 (16.0–18.0)	18.0 (16.0–20.0)	19.5 (17.0–23.0)	20 (18.0–22.0)	20.0 (18.0–25.0)	<0.01
Current daily smoker, *N* (%)	63 (28.0)	79 (17.1)	118 (19.9)	77 (13.0)	27 (4.8)	<0.01	97 (23.3)	93 (23.4)	78 (19.3)	67 (16.0)	36 (8.7)	<0.01
First cigarette within 30 min of waking, *N* (%)	24 (38.1)	35 (43.8)	46 (39.0)	27 (35.1)	5 (18.5)	0.22	44 (45.4)	43 (46.2)	33 (42.3)	34 (50.7)	15 (41.7)	0.86
Would like to quit smoking, *N* (%)	33 (64.7)	43 (62.3)	58 (59.8)	43 (70.5)	11 (61.1)	0.74	64 (55.2)	55 (53.4)	45 (50.6)	43 (58.1)	24 (58.5)	0.86

Results are number (%), or median (interquartile range). *N*—number.

**Table 4:** Proportion of daily smoking according to education, alcohol consumption, and physical activity, by gender and study year.

Variable	Men		*p*-Value Between Study Years	Women		*p*-Value Between Study Years
	2009 600 (43.6)	2019 725 (36.0)		2009 364 (15.0)	2019 371 (18.1)	
Education level, *N* (%)						
Primary	74 (42.0)	149 (55.0)	<0.01	25 (12.2)	67 (33.2)	<0.01
Secondary	389 (49.2)	496 (38.4)		236 (16.5)	236 (21.7)	
Higher	133 (34.5)	80 (17.7)		102 (13.2)	68 (8.9)	
*p*-value (difference in the same year)	<0.01	<0.01		0.06	<0.01	
Alcohol consumption, *N* (%)						
Never	260 (46.8)	104 (24.9)	<0.01	163 (14.0)	87 (14.9)	<0.01
≤once a month	193 (49.1)	219 (34.2)		126 (25.5)	147 (16.2)	
2–4 times a month	47 (50.0)	251 (39.7)		13 (31.0)	111 (23.7)	
2–3 times a week	27 (51.9)	119 (44.1)		4 (21.1)	24 (28.9)	
≥4 times a week	-	32 (58.2)		-	2 (25.0)	
*p*-value (difference in the same year)	0.80	<0.01		<0.01	<0.01	
Health evaluation, *N* (%)						
Good	296 (46.3)	393 (33.2)	<0.01	162 (16.8)	167 (17.5)	<0.01
Fair	262 (42.4)	299 (38.0)		178 (14.4)	160 (17.7)	
Bad	30 (39.0)	87 (42.0)		20 (12.8)	44 (22.2)	
*p*-value (difference in the same year)	0.24	0.02		0.19	0.28	
Physical activity, *N* (%)						
Inactive	NA	80 (39.8)	NA	NA	44 (16.5)	NA
Moderately active	NA	261 (31.4)		NA	199 (16.9)	
Active	NA	382 (39.0)		NA	128 (21.2)	
Professional athlete	NA	2 (33.0)		NA	0 (0.0)	
*p*-value (difference in the same year)	NA	<0.01		NA	0.10	

Results are number (%). NA—not available.

**Table 5:** Distribution of physical activity levels among daily smokers stratified by age and education in men and women.

Age Group	Education	Total in the Group (*N*)	Inactive, *N* (%)	Moderately Active, *N* (%)	Active, *N* (%)	*p*-Value
25–34	Primary	31	0 (0.0)	6 (19.4)	25 (80.6)	0.28
	Secondary	85	4 (4.7)	22 (25.9)	58 (68.2)	0.40
	Higher	8	1 (12.5)	5 (62.5)	2 (25.0)	0.25
35–44	Primary	61	2 (3.3)	16 (26.2)	43 (70.5)	0.21
	Secondary	85	3 (3.5)	16 (18.8)	66 (77.6)	<0.01
	Higher	23	2 (8.7)	14 (60.9)	7 (30.4)	0.48
45–54	Primary	13	1 (7.7)	6 (46.2)	6 (46.2)	0.80
	Secondary	131	10 (7.6)	48 (36.6)	72 (55.0)	0.48
	Higher	16	0 (0.0)	7 (43.8)	9 (56.3)	0.51
55–64	Primary	19	4 (21.1)	6 (31.6)	9 (47.4)	0.47
	Secondary	131	18 (13.7)	49 (37.4)	64 (48.9)	0.30
	Higher	19	2 (10.5)	10 (52.6)	7 (36.8)	0.77
65–74	Primary	25	9 (36.0)	10 (40.0)	6 (24.0)	0.54
	Secondary	64	19 (29.7)	38 (59.4)	7 (10.9)	0.03
	Higher	14	5 (35.7)	8 (57.1)	1 (7.1)	<0.01
25–34	Primary	18	0 (0.0)	9 (50.0)	9 (50.0)	0.08
	Secondary	58	1 (1.7)	33 (56.9)	24 (41.4)	0.57
	Higher	21	1 (4.8)	9 (42.9)	11 (52.4)	0.26
35–44	Primary	23	4 (17.4)	8 (34.8)	11 (47.8)	0.51
	Secondary	53	3 (5.7)	32 (60.4)	18 (34.0)	0.74
	Higher	17	0 (0.0)	10 (58.8)	7 (41.2)	0.13
45–54	Primary	13	0 (0.0)	10 (76.9)	3 (23.1)	0.07
	Secondary	48	8 (16.7)	24 (50.0)	16 (33.3)	0.12
	Higher	17	2 (11.8)	9 (52.9)	6 (35.3)	0.70
55–64	Primary	5	1 (20.0)	2 (40.0)	2 (40.0)	0.47
	Secondary	54	5 (9.3)	33 (61.1)	16 (29.6)	0.39
	Higher	8	3 (37.5)	4 (50.0)	1 (12.5)	0.18
65–74	Primary	8	4 (50.0)	3 (37.5)	1 (12.5)	0.75
	Secondary	23	9 (39.1)	11 (47.8)	3 (13.0)	0.16
	Higher	5	3 (60.0)	2 (40.0)	0 (0.0)	0.02

Results are number (%). *N*—number.

**Table 6:** Association between current daily smoking and independent factors for men and women in 2009 and 2019.

Variable	2009			2019		
Men						
	Odds ratio	95% confidence interval	*p*-value	Odds ratio	95% confidence interval	*p*-value
Age	0.97	0.96–0.99	<0.01	0.98	0.97–1.00	<0.01
Education	0.82	0.60–1.14	0.24	0.48	0.38–0.59	<0.01
Family status	0.97	0.79–1.20	0.78	1.23	1.11–1.37	<0.01
Smoking start	1.04	1.00–1.08	0.05	1.02	0.99–1.05	0.24
Alcohol consumption	0.71	0.58–0.87	<0.01	1.30	1.17–1.46	<0.01
Women						
Age	0.99	0.97–1.00	0.11	0.98	0.97–0.99	<0.01
Education	0.48	0.32–0.72	<0.01	0.39	0.29–0.50	<0.01
Family status	1.16	0.94–1.43	0.16	1.05	0.92–1.20	0.49
Smoking start	1.03	1.00–1.06	0.06	1.01	0.98–1.04	0.45
Alcohol consumption	1.06	0.77–1.47	0.72	1.05	0.88–1.25	0.60

**Table 7:** Sensitivity analysis of the association between current daily smoking and independent factors including physical activity (2019 data).

Variable	Men			Women		
	Odds Ratio	95% Confidence Interval	*p*-Value	Odds Ratio	95% Confidence Interval	*p*-Value
Age	0.99	0.98–1.00	0.01	0.98	0.97–1.00	0.01
Education	0.48	0.39–0.59	<0.01	0.38	0.29–0.50	<0.01
Family status	1.24	1.11–1.38	<0.01	1.05	0.92–1.20	0.48
Smoking start	1.02	0.99–1.05	0.25	1.01	0.98–1.04	0.43
Alcohol consumption	1.30	1.17–1.45	<0.01	1.04	0.87–1.24	0.66
Physical activity	1.07	0.90–1.28	0.43	1.09	0.84–1.40	0.53

Note: Physical activity was analyzed in three levels (inactive, moderately active, active), with professional athletes included in the active group.

## Data Availability

The data underlying this article will be shared on reasonable request to the corresponding author.

## Supplementary Materials

The following supporting information can be downloaded at: https://www.mdpi.com/article/10.3390/medicina62061201/s1. Table S1. STROBE Statement—Checklist. Figure S1. Direct Acyclic Graph for the association with current daily smoking.

## Abbreviations

The following abbreviations are used in this manuscript:

CI	Confidence interval
CVD	Cardiovascular disease
EU	European Union
IQR	Interquartile ranges
Max	Maximum
Min	Minimum
N	Number
NA	Not available
OR	Odds ratio
